# Engineering Biomimetic 3D Microenvironments for Extracellular Vesicle Programming Toward Clinical Translation

**DOI:** 10.3390/ijms27146121

**Published:** 2026-07-08

**Authors:** Ethan Nabeta, Andrew Wang, Junwei Zhao, Dake Hao

**Affiliations:** 1 Department of Surgery, University of California Davis, Sacramento, CA 95817, USA; enabeta@health.ucdavis.edu (E.N.); anbwang@health.ucdavis.edu (A.W.); 2 Shriners Children’s Northern California, Sacramento, CA 95817, USA; 3 Cosumnes Oaks High School, Elk Grove, CA 95757, USA; 4 Department of Biochemistry and Molecular Medicine, University of California Davis, Sacramento, CA 95817, USA; juwzhao@health.ucdavis.edu

**Keywords:** 3D microenvironments, biomechanical cues, biochemical cues, extracellular vesicle production, therapy

## Abstract

The cell secretome includes extracellular vesicles (EVs), nanoscale lipid bilayer-enclosed particles that carry diverse bioactive cargos, including proteins, lipids, and nucleic acids. As key mediators of paracrine signaling, EVs reflect the molecular and functional characteristics of their parent cells and play critical roles in regulating tissue homeostasis and regeneration. Growing evidence supports their therapeutic potential across a wide range of diseases. However, the clinical translation of EV-based therapies remains limited by challenges related to yield, purity, targeting specificity, and functional consistency. Recent advances in biomimetic culture systems—particularly three-dimensional (3D) platforms that recapitulate features of the native extracellular matrix microenvironment—have demonstrated a strong influence on cell phenotype, secretory activity, and EV composition. This review highlights how biochemical and mechanical cues within 3D culture systems regulate EV biogenesis, cargo loading, and functional outcomes and discusses their implications for improving the scalability, efficacy, and clinical translation of EV-based therapeutics.

## 1. Introduction

Extracellular vesicles (EVs) are nanoscale, membrane-enclosed particles secreted by virtually all cell types, including both eukaryotic and prokaryotic cells [1,2,3]. EVs play a central role in intercellular communication by transporting a diverse array of bioactive cargo, including proteins, lipids, and nucleic acids, between cells. In eukaryotic systems, EVs are primarily generated through two major biogenetic pathways. One pathway involves outward budding of the plasma membrane, which results in the direct release of vesicles into the extracellular space [1,2,4]. The second pathway is endosomal in origin, where inward budding of the plasma membrane leads to the formation of early endosomes that subsequently mature into multivesicular bodies (MVBs). These MVBs eventually fuse with the plasma membrane and release intraluminal vesicles—commonly referred to as EVs—into the extracellular environment [1,5,6]. Owing to their cellular origin, EVs inherit both membrane components and molecular cargo from their parent cells, thereby reflecting the physiological and functional state of the source cells [7].

As natural mediators of paracrine signaling, EVs possess a unique ability to cross biological barriers and deliver functional biomolecules to recipient cells, thereby regulating diverse cellular processes [1,2]. Compared with cell-based therapies, EVs offer several important advantages. They are non-replicative and inherently non-tumorigenic, reducing safety concerns associated with uncontrolled cell proliferation [8,9,10,11,12,13,14]. In addition, EVs exhibit low immunogenicity and toxicity, improved stability during storage, a prolonged shelf life, and greater flexibility in dosing and administration [15]. Their nanoscale size and lipid membrane structure further facilitate tissue penetration and efficient cellular uptake. Importantly, EVs can also be engineered through cargo loading or membrane modification to enhance their therapeutic potential [16,17,18,19,20,21,22,23,24]. Among the various cell sources investigated for EV production, mesenchymal stem cells (MSCs) have emerged as one of the most clinically relevant producers due to their multipotency, immunomodulatory properties, and regenerative capacity [25]. MSC-derived EVs have demonstrated promising therapeutic effects in a broad range of diseases, including musculoskeletal, cardiovascular, and neurological disorders [25].

Despite these advantages, the clinical translation of EV-based therapeutics remains constrained by several major challenges [26,27]. Conventional two-dimensional (2D) cell culture systems, which were initially used for EV isolation and production, are inherently limited in their ability to generate EVs at sufficient yield and consistency for large-scale applications. This is primarily due to their geometry and lack of laboratory space. In a single culture flask, for example, cells are provided with only one surface on which to grow. However, if a second flask whose exterior occupied the same amount of physical space as the first was given stacks of multiple interior layers for the cells to grow, then that second flask would naturally be able to house more cells that could also secrete more EVs. Like cell cultures in the laboratory, it is more efficient to plan a high-rise apartment complex in a crowded city that occupies half a block compared to a single-story complex that requires the entire block. The effectiveness of utilizing 3D culture vessels in improving EV yield has been shown [28]. One study even reported a 140-fold increase in EV yield from cells cultured in 3D vs. 2D environments [29].

In addition to low production efficiency, EVs derived from 2D cultures often exhibit variability in cargo composition and biological activity due to the oversimplified and artificial culture environment. These limitations highlight the need for advanced culture systems capable of more accurately recapitulating the complex cellular microenvironment found in vivo. In this context, three-dimensional (3D) cell culture platforms have emerged as a promising strategy to enhance both the quantity and quality of EV production [30,31,32,33,34,35,36]. Compared with traditional 2D culture systems, 3D platforms provide a biomimetic microenvironment that more closely resembles the native extracellular matrix (ECM), enabling improved cell–cell interactions [37,38,39,40,41,42,43,44], cell–matrix interactions [45,46,47,48,49,50,51,52,53], and exposure to physiologically relevant biochemical and biomechanical cues. These features help maintain native cell morphology, phenotype, and physiological function [54,55,56,57,58,59,60] while reducing the mechanical stress often associated with conventional culture methods and bypassing the risk of the development of spheroids that can form necrotic cores from which larger apoptotic bodies (ApoBDs) are released [61]. While, according to recent research, ApoBDs obtained from such 3D spheroid suspension cultures may hold some therapeutic value, slow clearance of them by an individual can result in tissue inflammation and other unwanted downstream effects [62], and the lack of specific cargo, as sorted from the Golgi Apparatus with the interplay between the endosomal sorting complex required for the transport (ESCRT) pathway [63,64], may diminish their potency. Consequently, 3D systems can significantly influence EV biogenesis, cargo loading, secretion dynamics, and overall therapeutic function. One study compared the proteome between EVs derived from human choriocarcinoma cells in 2D vs. 3D culture scenarios. In the study, it was revealed that 2D EVs were of equivalent sizes (between 100 and 200 nm) in fluorescent NTA measurements, shared similar expected morphologies according to transmission electron microscopy (TEM) images, and expressed the same classical EV markers (CD9, CD81, CD63, FLOT1, and TSG101). They expressed 57 unique proteins, and 3D EVs expressed 102, which leads to greater activation than suppression of multiple cellular pathways, according to Gene Ontology (GO) and Kyoto Encyclopedia of Genes and Genomes (KEGG) pathway analysis [61]. Importantly, the tunability of 3D culture systems offers unique opportunities to regulate cellular behavior and EV production through the controlled manipulation of environmental parameters, including matrix stiffness, topography, porosity, and biochemical signaling. Alterations in these physicochemical and biomechanical cues can modulate intracellular signaling pathways and gene expression profiles, leading to changes in cellular secretory phenotypes and EV composition. As a result, 3D biomimetic platforms not only improve EV yield but also enable the generation of EVs with enhanced or tailored therapeutic functions.

In this review, we discuss the fundamental rationale for developing 3D biomimetic culture systems for EV production, with a particular emphasis on how biochemical and mechanical cues regulate EV biogenesis, cargo composition, and functional outcomes. We further highlight recent advances in engineering strategies aimed at improving EV yield, quality, reproducibility, and scalability, as well as their implications for clinical translation. Finally, we discuss current challenges and future directions in the design of advanced biomaterial-based platforms to facilitate the widespread adoption of EV-based therapeutics.

## 2. Biomaterial Parameters Regulating Cell Behavior

The ECM is essential for proper embryogenesis and helps maintain stem cell pluripotency, quiescence, and the ability to survive, proliferate, migrate, and delay or advance differentiation [65,66,67]. It is these biological and mechanical factors of the ECM that drive such changes in cell behavior. Different signaling pathways and genes have long been known to be regulated through interaction with G protein-coupled receptors, receptor tyrosine kinases (RTKs), and nuclear hormone receptors [68,69]. Additionally, the formation of focal adhesions to activated integrin [2,65,70,71] triggers the opening of ion channels and cryptic peptide binding sites involved in the linker of the nucleoskeleton and cytoskeleton (LINC) complex, where sufficient tension can enable the transcription of various genes [68,72,73,74,75,76]. This system of physical mechanical cues initiating downstream responses describes a process known as mechanotransduction [77]. We will introduce how the interplay between biological and biomechanical factors affects cell behavior and EV production through simple receptor interaction and more complex mechanotransduction pathways. However, it must be recognized that the current literature in this field offers little in terms of how biomaterials specifically mechanistically regulate EV production and composition. Thus, based on the idea that EVs possess many of the functional properties of their parent cells, the information presented here regarding cellular changes is deemed relevant. In other words, cellular changes are likely to affect their respective EVs in a similar way, albeit indirectly. A representative but by no means exhaustive list of proteins and associated pathways commonly affected by the ECM includes integrin, FAK, MAPK/ERK1/2, Rho, ROCK, and YAP/TAZ. Typically, with the exception of a few relatively nondescript intermediate proteins, integrin stimulates FAK, which then typically activates MAPK/ERK1/2 [78] and leads to increased cell proliferation, survival, and differentiation or RhoA and ROCK, which facilitate actomyosin contraction [79,80,81] to open the LINC complex and allow YAP/TAZ to mediate the transcription of genes pertaining to cell proliferation and differentiation [82]. The significance of such proteins in specific studies will be further explored in the following sections. However, due to differences in cell source, integrin subtype expression, and ECM composition, among other factors, it is difficult to ascribe a universal outcome of cellular and EV changes based on the activation of these proteins to just one alteration of the 3D culture. The complex interplay between biochemically vs. mechanically activated pathways makes this task even more challenging. Much of the current literature also lacks the data to critically elucidate what triggers caused certain effects, which highlights the need for further studies and stricter standardization if developing therapeutic EVs is the ultimate goal. There exist such fine details in EV production that even certain studies comparing the functionality of 3D EVs have found lower potency compared to 2D EVs [61,83]. Therefore, with each section, we will attempt to synthesize the meaning and decouple the interdependency of the results on stiffness, pore size, patterning, and chemical signaling in order to highlight the effect(s) that each parameter has on an individual basis.

### 2.1. Stiffness

In terms of both the bulk strength of the matrix as a whole and the local stiffness of the matrix’s individual components, substrate stiffness is important in bearing physical weight [65,77,84,85,86,87] and aiding the stabilization of key cell-to-cell interactions [88,89]. In the case of viscoelastic substances like tissue ECM and some hydrogels, stiffness describes the substance’s resistance to an applied force, typically originating from compression, elongation, or shear stress sources, and is calculated by dividing this force by the substance’s strain as measured by deformation distance. Specifically, the Young’s modulus of stiffness, or elastic modulus, is a measure of a substance’s resistance to compressive (shrinking) or tensile (stretching) forces along the axis perpendicular to its surface. The shear modulus of stiffness is a measure of a substance’s resistance to forces parallel to its surface [90,91,92]. Conversely, the relaxation modulus describes the decrease in the elastic modulus over time, allowing the material to be strained more easily [93]. Elastic, shear, and relaxation moduli under the general topic of stiffness are important considerations for designing ECM mimics of native tissues, where each can differ drastically depending on their location and function within the body [94,95,96]. Different tissues such as adipose, liver, brain, coagulated bone marrow, initial fracture hematomas, and reconstituted ECM composed of collagen or fibrin have similar initial elastic moduli yet different rates of relaxation [92,97,98,99,100,101]. However, the ECM must have sufficient initial stiffness in order to direct cell behavior through mechanotransduction (Table 1). For example, Chaudhuri et al. found that murine MSCs seeded in hydrogels with initial elastic moduli of 9 kPa and slow relaxation rates were more adipogenic when compared to cells seeded in gels with elastic moduli of 17 kPa and rapid relaxation rates that were more osteogenic due to greater actin–myosin contraction [92]. Likewise, Hsieh et al. discovered that softer hydrogels promoted adipogenesis, in contrast to stiffer hydrogels, which promoted osteogenesis owing to the highly organized F-actin stress fibers within MSCs compared to softer hydrogels that promoted adipogenesis and diffused F-actin [102]. For both studies, it is likely that focal adhesions made between the cell and stiffer matrices were strong enough to reveal cryptic binding sites and enable Ras homolog gene family member A (RhoA) and RhoA kinase (ROCK) to stretch nuclear pores and allow an influx of Yes-associated protein, YAP, to activate the expression of osteogenic genes [2,103]. Another interesting study by Park et al. found that MSCs proliferate 30% more slowly on collagen-I-coated polyacrylamide gels with elastic moduli of 1 kPa compared to gels with elastic moduli of 3 and 15 kPa. However, cells seeded on softer hydrogels were also found to have fewer and weaker focal adhesions overall, which could explain the lack of phosphorylated extracellular signal-regulated kinase 1/2 (ERK1/2), which, along with YAP, helps drive cell proliferation [104,105,106]. However, the introduction of a signaling protein called transcription growth factor beta (TGF-β), as discussed in more detail in Section 2.4, altered the gene expression of the cells in spite of substrate stiffness, which implies that stiffness is not the only parameter that changes cell behavior [104].

### 2.2. Pore Size

In close relation to stiffness, it is also important to consider how the pore size of the ECM may affect cell behavior (Table 2), because different tissues also feature differences in pore size depending on the tissue function. Generally, larger pore sizes, such as those seen in cardiac and lung tissue, decrease matrix stiffness due to the absence of mechanically supportive components within the larger void spaces. Smaller pore sizes like those observed in skin and bone tissue, on the other hand, generally increase stiffness, as their tight packing and smaller void spaces provide greater mechanical strength [107,108,109,110]. Additionally, scaffolds of higher porosities (total void space) increase the overall surface area, which leads to the formation of more focal adhesions and the nuclear translocation of YAP to drive osteogenesis. This was demonstrated by Aarvold et al., where skeletal stem cells (SSCs) seeded on higher-porosity scaffolds saw higher alkaline phosphatase activity (ALP), a marker for osteogenesis, compared to cells seeded on lower-porosity scaffolds with equal pore sizes and stiffnesses [111]. As previously mentioned, osteogenesis could stem from the nuclear translocation of YAP, but other studies show that YAP’s influence may be context-dependent and have no effect, as shown by Chaudhuri et al., where both osteogenic and adipogenic cells saw equivalent levels of nuclear YAP [92]. Likewise, YAP might actually inhibit Runt-related transcription factor 2 (Runx2)—a key transcriptional factor for osteoblast differentiation [112,113]. However, a study led by Swanson et al. compared two porous scaffolds with similar porosities and stiffnesses but different pore sizes. The team found that murine brain cranial neural crest-derived cells, referred to as suture mesenchymal stem cells (SMSCs) and trunk-derived bone marrow mesenchymal stem cells (BMSCs), when adhering to scaffolds with larger pores, have a less extreme subtending angle compared to scaffolds with smaller pores that have a more extreme subtending angle. Such high curvature of smaller pores results in the phosphorylation of YAP, decreased cytoskeletal strain, and, ultimately, the maintenance of cell stemness and prevention of osteogenic differentiation through the prevention of the nuclear translocation of YAP [114]. Higher porosity and larger pores also allow cells to incorporate themselves immediately and proliferate within the matrix without having to first degrade and remodel it [110,115,116]. This aspect might have allowed Lowen et al. to find that human bone marrow-derived MSCs (hBMSCs) could form larger cell aggregates in scaffolds with larger pore sizes and smaller aggregates within smaller pores [115]. Pore size also influences cell morphology. For example, with the increased void space and decreased stiffness typically inherent in matrices with larger pores, cells tend to exhibit a rounder morphology and undergo adipogenesis or angiogenesis [25,108,117,118,119]. Using murine macrophages, Lowen et al. also found that, in addition to rounder morphology, larger pores allowed macrophage polarization towards the pro-regenerative M2 phenotype, while smaller pores led to elongated and spread-out macrophages with higher pro-inflammatory M1 populations [115]. It is also known that larger pores allow for more efficient nutrient transportation, migration, and vascularization, further facilitating cell growth [110,116,119,120]. Conversely, if a matrix is stiffer or has smaller pore sizes, it is more probable that a stem cell will assume a flatter or stretched morphology and undergo osteogenic or chondrogenic differentiation [121,122,123,124,125,126,127,128,129].

### 2.3. Patterning

As previously mentioned, cells are able to sense biomechanical cues from the environment and respond accordingly (Table 3). This includes being able to sense the topography or patterns in which they are seeded. For example, collagen fibers have an irregular arrangement in the dura mater membrane of the spinal cord, giving a relatively low elastic modulus with averages below 1 MPa [77]. However, this low stiffness is useful to confer a balance of flexibility and protection to this specific organ [130]. Additionally, myocytes are aligned uniaxially so that muscle systems may relax and contract high-tension forces on tendons that move bones. In vitro studies have recapitulated this directional motif by using grooved patterns to facilitate the lengthwise growth of myoblasts [131]—a result that, as Buskermolen et al. postulate, is likely due to the physics-based law of entropy and the natural tendency for cells to migrate from soft to stiffer substrates [132,133]. In parallel, a similar study by Gao et al. found that myoblasts undergo enhanced proliferation, myogenic differentiation, and myotubule alignment resulting from directional FAK and MAPK activation when seeded in patterned scaffolds featuring wider linear grooves compared to patterns with narrow linear grooves or smooth surfaces [134]. Additional studies have also shown the incredible potential of the trilineage differentiation of MSCs afforded by substrate patterning. Ferlin et al. found that, although both scaffolds were designed to exhibit equivalent porosity and pore volumes, hBMSCs seeded in scaffolds with cubical pores can drive the expression of adipogenic, chondrogenic, and osteogenic protein markers more effectively than scaffolds with cylindrical pores [135] These results, as Ferlin et al. hypothesized, might be a result of a higher magnitude of elastic moduli, pore sizes, and porosity uniformity permitted by the scaffolds with cubical pores [135], but the increased cell-to-cell interactions enabled by this scaffold design might have been crucial to maintaining the differentiation pathway of each lineage [136]. In another example, Muneekaew et al. found that seeding Wharton’s jelly mesenchymal stem cells (WJ-MSCs) on square microislands with or without extrinsic photobiomodulation (EPM) could drive adipogenesis and that WJ-MSCs seeded on rectangular patterns with EPM stimulation could drive neurogenesis, as EPM can trigger the release of Ca^2+^ that mediates differentiation [137,138,139]. However, the larger aspect ratio of rectangular patterns could have caused cells to stretch as more focal adhesions were formed [140,141], which could have resulted in an influx of Ca^2+^ as stretch-activated cation channels were opened [138] and ultimately result in neurogenesis, as shown in another study by Lee et al. [142]. On the single-cell level, Peng et al. demonstrate that MSCs exhibited increasingly greater osteogenic and less adipogenic differentiation with the secretion of ALP, an osteoblast indicator, or lipid droplets, an adipocyte indicator, when seeded on circular, square, triangle, and 5-point-star micropattern surfaces [143]. They hypothesize that the contrasts in differentiation pathways likely occurred because, compared to more angular shapes, the circular shapes were more evenly distributed, which also consequently resulted in decreased cellular traction forces [143]. However, the results of seeding multiple cells in 3D scaffolds do not always present themselves with clear mechanistic explanations. As Su et al. found, MSCs seeded in “mesh-like” patterned scaffolds demonstrate both round and stretched morphologies—shapes that were found primarily in randomly aligned scaffolds and aligned scaffolds, respectively. It could be, as the authors suggest, that the unique topography that helped define the cellular shape and mechanotransduction levels [140,144] also enhanced wound healing capabilities by altering paracrine signaling [145], which will be further explored in the next section.

### 2.4. Chemical Signaling

The biological properties of the ECM are equally as important as its structural properties [146,147]. To reiterate, when designing an ECM, the goal is to reflect or enhance the properties of the native tissue and the critical functions it encompasses [148,149]. To do so, we generally seek to find materials similar to the four main ECM protein classes: collagens, for their tensile strength and deformation resistance; glycoproteins that bind cell adhesion molecules like integrin and other ECM components; elastins, for their resiliency and ability to return to their original conformation after being stretched; and proteoglycans that bind water and provide compressive resistance [65,85,86,87]. However, these classes of macromolecules can have other unique layers of complex properties. For example, proteoglycans do provide compressive resistance and bind water, but their heparin sulfate side chains bind critical growth factors that influence cell fate [65,85,86,87,150,151]. Furthermore, different growth factors like vascular endothelial growth factors (VEGFs) and fibroblast growth factors (FGFs) have different affinities for particular heparin sulfate side chains, thereby contributing to the unique functional outcomes that vary from tissue to tissue [152,153]. For example, when FGFs are proteolytically released from the side chains, they can stimulate cellular signaling via RTKs and regulate cell growth, survival, and migration [65,69]. In fact, released FGF induces cell differentiation in the lungs and mammary glands [151,154,155,156]. Clearly, in addition to biomechanical mimicry, much consideration must be given to the biological aspect of the ECM, and many studies have needed hyaluronic acid [157], laminin [158], or chitosan [159] coatings in order to promote cell adherence and signaling [160]. The transmembrane protein receptor family is an especially popular choice for manipulating and examining the consequent effects, because there are 24 structurally and functionally distinct heterodimers formed by 18 different alpha and eight different beta subunits [161]. This diversity thereby allows for a myriad of outcomes when stimulated. One of the most common peptide ligands used to stimulate integrin proteins is arginine-glycine-aspartic acid (RGD), which is able to interact with eight out of the 24 integrin heterodimers [161,162,163,164]. It is found within the most common components of every ECM: collagen [165,166,167], gelatin [168], and the glycoprotein fibronectin [65,165]. In terms of functionality, RGD has been shown to enhance cell spreading [169], adhesion, and migration [115]. In fact, the study by Lowen et al. in Section 2.2 purposefully used RGD as a means to encourage cell adhesion and migration. With the combination of smaller void spaces, cell contact with RGD increased and led to cell spreading and lower M2 macrophage polarization, in contrast to scaffolds with larger void spaces and fewer RGD contact points, which led to the opposite results [115]. Likewise, in vivo, cells of various tissues mature and remodel the ECM through the secretion of matrix metalloproteinases (MMPs) that proteolytically degrade it [26,170,171]. For example, during angiogenesis, MSCs release hepatocyte growth factor (HGF), which stimulates the c-Met receptor tyrosine kinase in endothelial cells to prompt the cells to release vascular endothelial growth factor (VEGF) and MMP-1 to drive proliferation and migration into the ECM [172,173,174]. Such remodeling can also modify substrate stiffness, and softer matrices with more accessible surfaces potentially make it quicker and easier for additional interactions and the formation of focal adhesions to occur, including the accompanying changes related to cell survival, spreading, proliferation, migration, and differentiation [92,175,176,177]. Chaudauri et al., too, attributed osteogenic differentiation to the combination of high initial stiffness, which enables stronger actin–myosin-related tension, and the rapid relaxation of the gels; this combination facilitates more integrin binding to RGD sites [92]. Aside from RGD and VEGF, transforming growth factor beta (TGF-β), used to phosphorylate or activate Smad2/3 transcription factors for gene regulation [178], was used in the same study by Park et al. in Section 2.1, where it was found to increase the expression of smooth muscle cell markers (α-actin and calponin-1) for cells seeded on stiffer gels and increase the expression of chondrogenic marker (collagen-II) and decrease adipogenic marker (lipoprotein lipase or LPL) expression for cells seeded on softer gels [104]. While TGF-β signaling and mechanotransduction certainly impacted the differentiation pathways in that study, the exact mechanisms for such results are still unclear. To elaborate, RhoA activity that is normally involved in stress fiber formation and mechanotransduction was not found to change between soft and stiff gels, which indicates that, perhaps, Rho is constitutively expressed to maintain the expression of other unrelated genes. However, the antibody blocking of integrin for cells seeded on stiff substrates appeared to mimic the effects of softer gels: it decreased smooth muscle cell markers and increased chondrogenic and adipogenic markers [104]. Likewise, the use of laminin-coated substrates by Lee et al. resulted in the neurogenic and adipogenic differentiation of adipose tissue (AT-MSCs) that had a higher expression of αv and β3 integrin subunits and bone marrow (BM-MSCs) that had a higher expression of α1, α5, α6, and β1 integrin subunits, respectively [142]. Altogether, these results demonstrate the importance of both biomechanical and biochemical cues in directing behavioral changes in specific cell types (Table 4).

## 3. 3D Scaffold Platforms for Regulating Cellular and EV Responses

**Figure 1:**
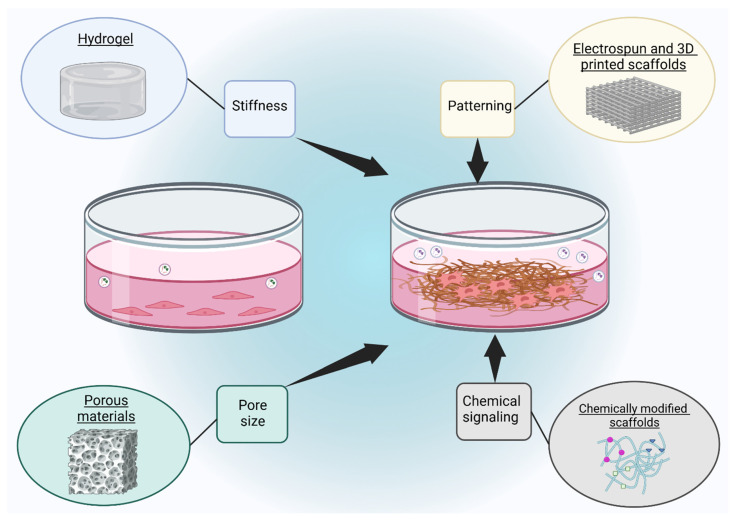
Schematic illustration of biomaterial parameters and scaffold platforms regulating cellular and EV responses. Created in https://BioRender.com.

As previously discussed, the native ECM is the ideal structure for culturing cells and driving their proliferation, migration, and differentiation [179]. Hence, the biological and mechanical factors of ECM have guided the advent of unique 3D scaffolds to accommodate the specialized regulation of cell behavior and EV production (Figure 1).

### 3.1. Hydrogel

Hydrogels made of natural or synthetic polymers are primarily hydrophilic and expand when they retain water. They are also functionalized with at least one additional component: a crosslinking peptide to bind the polymer chains together to create the matrix network. As they absorb more water and their volume swells, they assume the shape of their vessel. Some hydrogels also incorporate hydrophobic polymers to imbue water-resistant properties that can resist the adhesion of certain unwanted bacteria and algae [180]. Hydrogels can also self-heal after physical disruptions, depending on covalent, noncovalent, and physical bonding interactions. For these reasons, these scaffolds and the materials that comprise them find applications in cell culture, tissue engineering, biosensors, hydrophobic drug delivery, and 3D printing, the latter of which will be discussed further in Section 3.3 [181] (Table 5). Typically, hydrogels are also relatively simple to produce, and their stiffness, which plays a crucial role in cell behavior, is easy to modify, which makes them a popular choice for initial cell cultures. The stiffness of hydrogels is primarily modified by changing two parameters: the crosslinkers and the efficiency with which those crosslinkers bind. For instance, crosslinkers possessing various chemical functional groups, densities, and molecular weights may be added to hydrogel mixtures and bind to the arms of the base polymer material at faster or slower rates depending on reaction conditions like pH, temperature, light wavelength, and incubation time [77,182]. Altering the molecular weight of the base polymer to increase entanglement and binding interactions [183,184,185] and the addition of nanoparticles such as nanosilicate, whitlockite (WH), hydroxyapatite (HAP), and graphene oxide (GO) to further interact with and reinforce polymer chains can also increase stiffness [186,187,188,189,190]. Lenzini et al. increased the stiffness of alginate hydrogel with the addition of extra calcium ions to form physical crosslinks or adipic acid dihydrazide (AAD) to form covalent crosslinks and found that human bone marrow aspirate MSCs seeded on softer hydrogels produce two-fold and five-fold more EVs per cell compared to cells seeded on stiffer hydrogels (~20 kPa) and rigid polystyrene plastic culture dishes, respectively. The EVs even retained the same size, morphology, and surface protein makeup across all stiffnesses. However, at equal doses, EVs isolated from murine MSCs seeded on soft hydrogels were able to reduce lung edema and vascular permeability more effectively compared to EVs from cells cultured on plastic substrates [191]. Hsieh et al. profiled the genetic expression of human MSCs (hMSCs) within polyacrylamide microgel scaffolds whose stiffnesses were increased with higher quantities of acrylamide monomer and bis-acrylamide crosslinker. The study found that increased stiffness, or storage moduli as measured through shear stress measurements, could induce cells to express a higher amount of osteogenic messenger RNA (mRNA) such as Runx2, osterix, type I collagen, ALKP, and osteocalcin, where stiffer matrices led to the more rapid maturation of osteoblasts, as evidenced by a decrease in Runx2 expression after 21 days for cells seeded in stiff hydrogel compared to cells seeded in softer hydrogels [102]. Although that study did not isolate EVs for analyses or functional tests, the findings could prove useful nonetheless, because, as mentioned, EVs retain much of the same characteristics as their parent cells [7]. Meaning, if the parent cell behavior changed toward therapeutic treatment improvements, then the EVs should improve as well. However, studies incorporating stiffness within their cell scaffold considerations also include pore size, because softer hydrogels generally have larger pore sizes. However, naturally porous materials themselves have also provided a few keen insights into cellular behavior and their reciprocal EV characteristics.

### 3.2. Porous Scaffolds

Scaffolds with an emphasis on porosity can be made via emulsion freezing, particulate leaching, solvent casting, emulsion templating, gas foaming, melt molding, or 3D printing [192,193] (Table 6). The latter method has been used in conjunction with bioactive ceramic materials that can optimize pore sizes for cell culture or implantation. One of the most common bioactive ceramic materials is the nontoxic and antibacterial composite known as bioglass, of which three main types exist, whose main components are silicate, phosphate, and borate [194,195,196]. Printing a bioglass scaffold defined by the user via 3D modeling computer software involves glass powder and binder, which are sintered or heated just before melting to allow for compaction and formation of the shape and pore designs [197]. Cubical, spherical, crossed, gyroid, diamond, and potentially more pore geometries are possible [198]. Additionally, melt-quenching and sol-gel processing methods enable bioglass microsphere production, which could prove a more effective form factor as an injectable medium for a less invasive therapy compared to surgical methods [199,200]. Aside from its fabrication aspects, when bioglass degrades, it releases silica, calcium, salt, and potassium that encourage osteogenesis and angiogenesis [196]. Another relevant material used for making porous scaffolds is hydroxyapatite (HA), which is crystallized calcium phosphate—a mineral found within bone that can support MSC proliferation, migration, and osteogenesis [103,201]. One study by Lian et al. used copolymer poly (l-lactic acid-ε-caprolactone) PLCL and HA nanoparticle components to compare the effects of paracrine signaling from rat BMSCs after seeding them into two different scaffolds. The fibers of one scaffold were smooth, as the base components were heat-molted together at 200 °C. The other scaffold, however, simply dissolved the base components at room temperature and collected the solution on a cryogenic collector plate at −28 °C to allow the formation of micropores on the fibers. These micropores enabled greater cell adhesion, spreading, and proliferation and more effective paracrine secretion of angiogenic, osteogenic, and immunomodulatory factors, including those that promote the M2 macrophage polarization of RAW264.7 macrophages. MSCs seeded in these microporous scaffolds were also more effective at promoting vascularized bone regeneration in rat distal femoral defect models [202]. In two parallel studies by Swanson et al., as mentioned in Section 2.2, poly (L-lactic acid) (PLLA) scaffolds were crafted by emulsifying sugar within oil, treating with different temperatures to generate different pore sizes, then freeze-drying the dissolvable sugar to form a foundation for the PLLA scaffold [203]. When these scaffolds were used to seed murine BMSCs and suture mesenchymal stem cells (SMSCs), those with smaller pore sizes led to a lower expression of osteogenic markers CTGF, YAP1, CD146, Runx2, and SP7, with a higher expression of Col3 and SMSC marker Gli1. Opposite results were seen for scaffolds with larger pores [114,204]. The study by Lian et al. as well as a few other authors in this review, on the other hand, utilized 3D printing for scaffold construction, which will be discussed next.

### 3.3. Electrospun and 3D Printed Scaffolds

Aside from weaving polymer fiber meshes and strengthening their bonds, phase separation techniques, and molecular self-assembly in the case of bulk hydrogel formation, we have seen that the advent of electrospinning and computerized 3D printers provides two of the most precise methods for crafting custom patterns for scaffolds with unique stiffnesses, pore sizes, and patterning [192,205,206] (Table 7). 3D printers utilize an additive manufacturing approach to scaffold building, where each new layer of biomaterials is constructed or extruded from the printer head and onto the previous layer until the job is complete [207]. Generally, 3D printers have a high degree of control over the overall scaffold shape but low nanofiber resolution. Like 3D printers, electrospun scaffolds are also easy to fabricate but offer precise control over parameters like patterning and fiber diameter rather than scaffold shape [208,209,210,211]. For example, greater electrical field strength and polarity via decreased distance between the needle and collector and faster fiber collection platforms generally lead to smaller fiber diameters, which then, along with greater fiber alignment, help to increase bulk scaffold stiffness [77,212,213]. In several studies, scientists have used such electrospun scaffolds to demonstrate encouraging signs of cell proliferation and maintain characteristics of stemness [214,215,216,217,218]. Specifically, depending on the fiber alignment, they can promote Schwann cell maturation, endothelial cell growth, myotube formation, and MSC differentiation [144,219,220,221,222]. One study utilized a scaffold constructed with polyacrylonitrile (PAN), a piezoelectric polymer capable of converting mechanical energy to electrical charge, to stimulate HepG2 cancer cells and 3T3 fibroblasts. For both cell types, it was found that EV production increased as a result of acoustic stimulation. However, a decrease in heat shock protein 90 (HSP90) was also observed, potentially leading to alterations in target cell protein folding, cancer progression, and wound healing [223]. The Ad-MSCs seeded in electrospun polycaprolactone (PCL) scaffolds in Su et al. in Section 2.3 were able to secrete paracrine factors (PGE2, iNOS, TGF-β, VEGF, bFGF, and HGF) that were more conducive to angiogenesis or the proliferation and tube formation of human umbilical vein endothelial cells (HUVECs) and the M2 polarization (IL-10 and Arg-1 secretion) of RAW264.7 macrophages. The team used a flat plate, a rotating cylinder, and copper mesh with different collection distances to craft scaffolds with random, aligned, and mesh fibrillar patterns, respectively. The mesh pattern provided the optimal pattern for such results, and the conditioned media from the seeded cells even led to neater collagen deposition at wound sites compared to media from cells seeded in random or aligned patterns [145]. Likewise, Liu et al. demonstrated that aligned polydimethylsiloxane (PDMS) electrospun fibers could induce human umbilical vein endothelial cells (ECs) to secrete EVs with higher levels of miR-143 and miR-145 more effectively than random fibers, and the authors suggested that these micro-RNAs (miRNAs) could maintain an atheroprotective SMC phenotype in future studies [224].

### 3.4. Scaffolds with Additional Chemical Signals

In Section 2.4, we saw that biomaterials could be functionalized with RGD to interact with integrin to enhance cell adhesion [92,115] and TGF-β to stimulate kinase receptors that activate certain genes for myogenic, chondrogenic, or adipogenic differentiation [104,225]. Perhaps such signals can modulate the cellular secretome as well. Both soft and stiff hydrogels designed by Lenzini et al. saw a two-fold increase in the amount of EVs released per bone marrow aspirate hMSC when the hydrogels were functionalized with five times less RGD or treated with cilengitide—a drug that interferes with RGD binding to integrin. The authors suggested that actin fibers pose a physical blockade that prevents EV secretion. To briefly expand on this idea, as cells attach to substrates via focal adhesions, focal adhesion kinase (FAK) and actin-related protein 2/3 (Arp2/3) are activated and promote the formation of actin fibers involved in cell spreading and migration [191,226]. However, such fibers subsequently act as a barrier to MVBs and prevent them from fusing with the plasma membrane and releasing their EVs [191]. After treating the MSCs with CK869, an Arp2/3 inhibitor, phalloidin-stained F-actin decreased and EV secretion increased in both soft and stiff hydrogels. Additionally, the knockdown of FAK activity via small interfering RNA (siRNA) increased EV secretion in cells seeded in stiff hydrogels. Another study found that the inhibition of integrin, ITGA1 in the case of human umbilical cord MSCs, subsequently led to the inhibition of the RhoA/cofilin signaling pathway involved in actin depolymerization. Such inhibition promoted EV release, with a yield approximately 2.5x higher for EVs isolated from 3D cultures compared to EVs from 2D cultured cells. EV sizes and morphologies remained the same across both groups as well [227]. These results lend credibility to the hypothesis that increased integrin binding, focal adhesions, and actin polymerization indeed prevent EV release [191]. The effects of chemical signaling on EV secretion and functional outcomes are well worth investigating, especially considering that minor alterations such as RGD concentration can double the number of EVs and can maintain high therapeutic efficacy, possibly through the actions of mRNAs (KGF and IL-6), miRNAs (miR-146A, miR-30b-3p, and miR-27a-3p), and even mtDNAs (ND1 and ATP6), as one study demonstrated with reductions in lung edema and vascular permeability in murine models of acute lung injury [191]. Of course, many other chemical signals exist. For example, 3-isobutyl-1-methylxanthine molecules can promote the neurogenic differentiation of cord blood mononuclear cells (MNCs) [228]. Moreover, growth factors in the VEGF, FGF, and HGF families are considered to be the main players that drive and maintain physiological angiogenesis [172], but their complexity and points of mechanistic intersection stress the importance of considering the full dynamic picture of pathway regulation [229]. Du et al. cultured human placenta-derived MSCs (hp-MSCs) with a nitric oxide-releasing chitosan polymer (CS-NO) and found that EVs secreted from these cells, compared to EVs secreted from cells cultured in control media, presented greater angiogenic potential when treating HUVECs and murine hind limb ischemia models. Importantly, the study found that proangiogenic VEGF and miR-126 upregulated in cells exposed to CS-NO were also found within the secreted EVs [230]. Because EVs maintain therapeutic efficacy and cargo when their parent cells are exposed to different chemical signals, they are being continuously studied as treatment options for several diseases that will be explored next (Table 8).

## 4. Bioengineered EVs as Delivery Vehicles

Recall that EVs play a central role in cell communication and are an ethically moral tool for studies involving tissue regeneration, drug delivery, and immunomodulation [231,232,233,234], because they are non-tumorigenic [8,9,10], stable, low in toxicity and immunogenicity [15], and easily modifiable for specific cargo and accurate dosages [16,17,18,19,20,21,22,23,24]. The intralumenal environment of EVs helps to preserve cargo, and their membrane composition facilitates easier passage through a target cell’s selectively permeable membrane. The properties that enable EV-cell fusion also prevent EVs from triggering an immune response and being cleared from the body. Thus, EVs are excellent delivery vehicles for functional proteins, RNA, and drugs and are useful in the myriad therapies (Table 9). Popular methods for loading EVs with such materials include electroporation [16,17,18], incubation [19,20,29], sonication [21,22,23], and nanoparticle-induced drug loading, where the latter’s use of graphene quantum dots has enabled up to 66% loading efficiency [24]. Now, each subcategory will be discussed in more detail.

### 4.1. RNA Delivery

Many types of RNA exist, but small RNAs (sRNAs) like siRNA and miRNA are the current focus as far as EV cargo that blocks the activation of other proteins is concerned. Via injection, custom naked DNA plasmids absorbed by the liver and processed into anti-H19 sRNA and packaged into EVs were able to enter the bloodstream and target and enter colorectal cancer cells and inhibit H19 long non-coding RNA (lncRNA) involved in carcinogenesis, progression, and metastasis more effectively than 5-Fu—a common anticancer drug [235]. Other siRNA molecules delivered by EVs have also been used to inhibit inflammation by targeting nuclear factor kappa B (NF-κB) in skin lesions and LPS-induced acute lung injury mouse models [236,237]. By tail vein injection of EVs packaged with α-Syn siRNA, Cooper et al. demonstrated the ability of EVs to cross the blood–brain barrier (BBB) to decrease α-Syn aggregates—indicators for the progression of Parkinson’s disease [238,239]. Through trickier and more invasive means, by injecting EVs loaded with the miRNA known as miR-124-3p, dopaminergic neurons within the substantia nigra and striatal fibers were protected and motor functions were normalized in mice modeling Parkinson’s disease [240]. On the other hand, mRNA has been utilized to translate proteins of interest that are also beneficial in EV-based disease treatments. However, this process may require puromycin treatment beforehand to prevent the premature translation of mRNA plasmids before they can be packaged within EVs [241]. Freeze-thawing may also be used to encourage the fusion of liposome-based content with EVs, as Wu et al. achieved with ALKBH5 mRNA to inhibit colorectal cancer progression [242]. Flag-tagged EV sorter protein designed to bind to the stem loop of an mRNA aptamer presents a means to produce and isolate mRNA-containing EVs. One such study utilized this method and anti-Flag magnetic beads to capture the Flag-EVs containing the mRNA recognized in treating atherosclerosis and other diseases [243]. Next, we will discuss the overlapping application of EVs as delivery vehicles of proteins.

### 4.2. Protein Delivery

Similar to the delivery of genetic materials, EVs loaded with functional proteins to deliver to target cells can be a tremendous advantage over delivering proteins alone. For example, Han et al. were able to reduce inflammation in lung epithelial cells by transfecting them with CC16, whereby their EVs were more effective in protecting against lipopolysaccharide (LPS)-induced lung injury by inhibiting reactive oxygen species (ROS), DNA damage, and inflammation compared to treatment with recombinant CC16 that was 1000-fold higher in concentration [244]. Like DNA and RNA, the exterior of proteins is hydrophilic, preventing their passage through the hydrophobic center of target cells’ lipid bilayer, and, as previously alluded to, without EV vehicles to seamlessly contact and fuse with cell membranes and stabilize cargo integrity, the likelihood of targeting, delivering, and ultimately exerting a functional effect on a specific cell to trigger a signaling cascade decreases [245,246,247,248]. Tetraspanins, integrins and immunoglobulins, proteoglycans, and lectins are all proteins, the blocking of which may also prevent EV signaling activation and uptake [249]. Because direct loading methods of EVs with proteins often lead to suboptimal refolding rates, stability, and activity, a relatively new strategy to load proteins into EVs emerged, where host cells could be transfected with plasmids containing the gene of interest so that it may be overexpressed and packaged naturally within their EVs, as Han et al. and others in the previous section accomplished [241,243,244,245,250]. Some researchers have even delivered multiple different plasmids to single host cells in order to overexpress and package multiple proteins within EVs to increase therapeutic efficacy [251,252] or have conjugated combinations of proteins and drugs to EVs to increase targeting capabilities [253].

### 4.3. Drug Delivery

In the delivery of highly potent drugs, the value of the specific targeting capabilities of EVs cannot be understated. When there is high specificity for diseased cells, fewer negative side effects as a result of targeting healthy cells occur. However, EV membranes must often undergo additional engineering and protein modifications to augment this characteristic. Some lipophilic drugs may be incubated with and loaded into EVs via passive diffusion through the membrane, but both lipophilic and more hydrophilic drugs can be loaded via incubation with detergents, sonication, heat shock, or electroporation to create porous EV membranes that allow drug entry. However, these methods are apt to damage EV membranes at the expense of higher loading efficiencies and precision [250,254,255]. In fact, Rehman et al. incorporated protein, RNA, and drug content into anti-cancer EVs [256]. The drug of choice for treating glioblastoma brain cancer was temozolomide (TMZ), which methylates guanine nucleotide bases on DNA to induce strand nicks and overwhelm the cell’s DNA repair mechanisms, ultimately leading to apoptosis [257]. Cancer aggression in mice implanted with TMZ-resistant glioblastoma decreased when the team sonicated EVs to incorporate TMZ and siSTAT3 into the EV lumen. This siSTAT3 is an siRNA used to inhibit the translation of STAT3—a cancer progression protein that is also responsible for TMZ resistance. The treatment was also more effective thanks to the integration of heme oxygenase-1 (HMOX1)-specific short peptide (HSSP) within the EV membranes to target drug-resistant cancer cells expressing HMOX1 [256]. Other studies have utilized EVs loaded with drugs that are activated by light [258,259] or sound [260,261,262,263,264] energy, which can provide highly specific and effective and minimally invasive cancer treatments.

**Table 9:** Representative cargoes delivered by EVs for therapeutic applications.

Cargo Type	Representative Cargo	Major Function	Representative Application	Reference
siRNA	Anti-H19, NF-κB siRNA, α-Syn siRNA	Gene silencing	Cancer, inflammation, Parkinson’s disease	[235,236,238]
miRNA	miR-124-3p	Post-transcriptional regulation	Neuroprotection in Parkinson’s disease	[240]
mRNA	ALKBH5 mRNA, therapeutic mRNA	Protein translation	Cancer, atherosclerosis	[242,243]
Proteins	CC16, targeting proteins	Anti-inflammatory signaling; targeted delivery	Lung injury, cancer	[244,253]
Drugs	TMZ, photosensitizers, sonosensitizers	Chemotherapy; photo-/sono-dynamic therapy	Glioblastoma, solid tumors	[256,259,260]
Combination cargoes	Drug + siRNA; protein + drug	Synergistic therapy	Drug-resistant cancer	[253,256]

## 5. Bioengineered EVs as Therapeutic Agents

**Figure 2:**
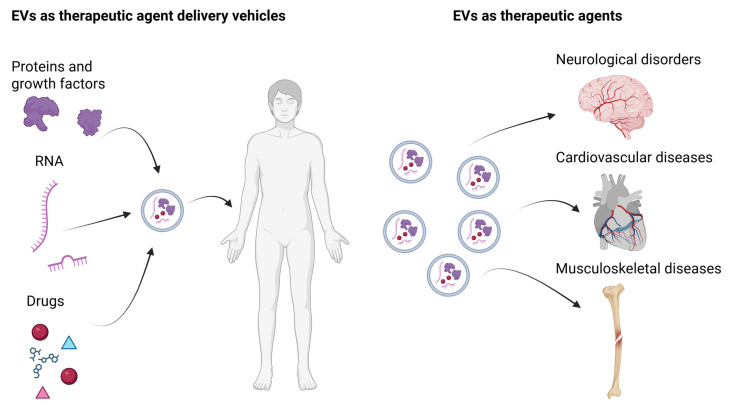
Clinical potential of bioengineered EVs as cargo delivery vehicles and therapeutic agents.

EVs have a wide and often overlapping array of uses, including medical therapies. EVs have seen much use in therapy, because the EVs themselves are non-toxic [15], relatively easy to handle and modify [16,17,18,19,20,21,22,23,24], and bypass the need for engraftment. Moreover, EVs have been shown to cross cellular, intracellular, and tissue barriers, including the blood–brain barrier (BBB), which makes them a highly safe, versatile, and efficient treatment option [238,265,266]. They can support additional EV biogenesis via positive feedback loops [231,267,268], cell adhesion [269,270,271], proliferation, migration [271,272,273], and immunomodulation [271,273]. Importantly, common denominators of therapeutic EVs include the ability to reduce apoptosis and inflammation and increase cell recruitment and angiogenesis. These properties and functions are necessary for their use in tissue regeneration and drug delivery in treating various diseases (Table 10). So far, they have been used as diagnostic markers, and we will also discuss their therapeutic use in treating neurological disorders, bone defects, and cardiovascular diseases (Figure 2).

### 5.1. Neurological Disorder Therapy

Neurological disorders include strokes, traumatic brain injury, spinal cord injuries, peripheral nerve injuries, epilepsy, Alzheimer’s disease, Parkinson’s disease, amyotrophic lateral sclerosis, multiple sclerosis, and Huntington’s disease, which are characterized by increased inflammation, neuronal apoptosis, toxic proteins, and leakage of the BBB [8]. With administration via systematic methods or nasal spray of therapeutic EVs derived from MSCs, ASCs, neurons, endothelial cells, endothelial progenitor cells, microglia, blood serum, cerebrospinal fluid (CSF), neurons, neural stem cells (NSCs), Schwann cells (SCs), M2 macrophages, olfactory ensheathing cells (OECs), pericytes, dental pulp stem cells, human brain microvascular endothelial cells (HBMVECs), stem cells from human exfoliated deciduous teeth (SHEDs), astrocytes, fibroblasts, and periodontal ligament stem cells, improvements can be made to a patient’s infarct size, hematoma clearance, brain edema, and neurological functions [8,173,274,275,276,277,278]. Interestingly, although smaller EVs are generally more therapeutic than larger EVs, larger EVs like microvesicles (MVs) can deliver organelles like mitochondria that could be particularly beneficial in treating victims of post-ischemic stroke [279,280]. As Dave et al. showed using human brain microvascular endothelial cells (hCMECs), isolating the associated large EVs (~185 nm average diameter) that contained mitochondria and likely mitochondrial DNA and proteins to treat human brain ECs contributed to increased mitochondrial function, which led to increased ATP levels. The additional ATP, therefore, promoted the survival of brain ECs and reduced brain infarct sizes in murine stroke models [280]. Likewise, Leggio et al. utilized EVs isolated from ventral midbrain–striatal astrocytes, known to deteriorate and die in individuals with Parkinson’s disease, and found that they could counteract activated caspase-3 to prevent apoptosis in differentiated but not undifferentiated SH-SY5Y neuroblastoma cells. Furthermore, for the differentiated cells, EVs from ventral midbrain astrocytes rescued ATP production [281]. In another study by Cone et al., EVs from human bone marrow MSCs were able to decrease extracellular amyloid-beta plaques in the 5XFAD murine model of Alzheimer’s disease [282]. Unfortunately, these last two studies only targeted universal EV markers for quantification and unique cargo, so the direct cause-and-effect relationship between EV treatment and neuroprotection [281] or amyloid-beta plaque reduction [282] cannot be conclusively stated.

### 5.2. Cardiovascular Disease Therapy

Cardiovascular diseases encompass heart failure, arrhythmias, strokes, and coronary artery disease, which are caused by atherosclerosis in the blood vessels [283,284]—a buildup of plaque (smooth muscle cells, macrophages and other white blood cells, collagen, glycosaminoglycans, elastin, fibrinogen, lipoproteins, lipids, and cholesterol) [285,286,287]. Luckily, such diseases can be treated with EVs [288]. Within the cardio system, including the heart, muscle, bone marrow, blood, and the umbilical cord, one may obtain EVs from endothelial cells (EDEVs), platelets (PEVs), vascular smooth muscle cells (SMCEVs), cardiomyocytes (CMEVs), MSCs, hematopoietic stem cells, cardiac progenitor cells, cardiospheres, and embryonic stem cells [288]. As these EVs can act locally or enter the bloodstream to act on distant cells from different organ systems, their effects are far-reaching and, hence, they have been used to regulate cardiovascular function, vascular tone and blood pressure, inflammation, tissue repair, and blood clotting [288,289]. In particular, EVs from mouse bone marrow-derived MSCs carrying miR-182 were able to polarize M1 macrophages to the M2 phenotype and reduce the inflammation and myocardial ischemia/reperfusion infarct size of murine models by inhibiting toll-like receptor 4 (TLR4) to increase the activation of the PI3K/Akt signaling pathway in a study helmed by Zhao et al. [290]. The effect that miR-182 had on macrophage polarization and inflammation reduction was also documented by Liu et al. [291]. Another study by Alexandru et al. demonstrated that EPCs from healthy control hamsters secreted EVs enriched in miR-10a, miR-21, miR-126, miR-146a, and miR-223, which activated insulin-like growth factor 1 (IGF-1) to stimulate the migration of endothelial cells for the repair of vascular injury in late EPCs from hypertensive–hyperlipidemic (HH) atherosclerotic hamster models [292].

### 5.3. Musculoskeletal Disease Therapy

Treating bone-associated defects such as arthritis and osteoporosis, like any tissue regeneration therapy, requires angiogenesis or the formation of blood vessels to osteogenic-supporting cell types through plasma carrying oxygen-rich blood cells and other nutrients. The process of osteogenesis involves osteoclasts’ removal of weak fibrous bone, chondrocytes laying cartilage foundation, and osteoblasts replacing it with strong lamellar bone [10,293,294]. Many bone defect treatments have utilized EVs from MSCs, immune cells, osteoblasts, and endothelial cells [295,296,297,298]. For example, bone marrow mesenchymal stem cells (BMMSCs) [299], adipose-derived mesenchymal stem cells (ADSCs) [300,301], umbilical cord mesenchymal stem cells (UCMSCs) [302,303], synovial MSCs [304], MSCs derived from human-induced pluripotent stem cells (hiPSCs) [305,306], and SHEDs [307,308] contain cargo that enhances macrophage polarization [291], osteogenic differentiation [291,309], and angiogenesis [310,311,312,313,314,315,316,317,318] and suppresses osteoporosis [304,319,320]. In one example, Mi et al. isolated EVs secreted by murine vascular ECs and loaded them with osteogenic miR-26a-5p to increase osteoblast differentiation and inhibit osteoclast differentiation [309], the regulation of which is important for bone remodeling and preventing osteoporosis [321]. Likewise, Liu et al. showed that the miR-146a cargo of rat BMSCs could enhance angiogenesis in developing zebrafish by targeting angiogenic inhibitors (Smad4 and merlin (NF2)). The increase in angiogenesis also contributed to the increase in osteogenesis in rats with distal femur defects [312]. Even EVs secreted by monocytes, dendritic cells, and M2 and naïve M0 macrophages can promote osteogenic differentiation [322] and immunomodulation [296] and inhibit osteoclast formation [323]. Osteoblast EVs contribute to the process of mineral deposition during the endochondral ossification of growing embryos, where bone tissue replaces cartilage [297,324,325]. Also, endothelial cell EVs suppress the differentiation and activity of osteoclasts [298] and promote angiogenesis and osteogenesis [326]. Lastly, chondrocytes and chondrogenic progenitor cell EVs help promote chondrocyte proliferation and migration [327] and the differentiation of BMMSCs into additional chondrocytes [328]. Wang et al. showed that EVs isolated from MRL/MpJ “superhealer” mice MSCs could improve the proliferation and migration of chondrocytes in osteoarthritic mouse models, likely due to the enhanced levels of miR-221-3p, which decreased these effects when inhibited [327].

**Table 10:** Therapeutic potential of EVs across various diseases.

Disease Model	EV Source	Functional EV Cargo	Therapeutic Effect	Reference
Stroke	Human brain microvascular endothelial cells (hCMECs)	Mitochondria	Increased survival of brain endothelial cells and reduced brain infarct sizes	[280]
Parkinson’s disease	Ventral midbrain–striatal astrocytes	Not reported	Neuroprotection of undifferentiated SH-SY5Y neuroblastoma cells	[281]
Alzheimer’s disease	Human bone marrow MSCs	Not reported	Reduction of extracellular amyloid-beta plaque	[282]
Myocardial ischemia/reperfusion (mice)	Murine bone marrow-derived MSCs	miR-182	M2 macrophage polarization and reduced inflammation and infarct size	[290]
HH atherosclerosis	Hamster EPCs	miR-10a, miR-21, miR-126, miR-146a, and miR-223	Enhanced IGF-1 activation and improved repair of endothelial vasculature	[292]
Femur bone defect	Rat BMSCs	miR-146a	Enhanced angiogenesis and osteogenesis	[312]
Osteoarthritis	MRL/MpJ mice MSCs	miR-221-3p	Enhanced proliferation and migration of chondrocytes	[327]

## 6. Discussion and Conclusions

In this review, we discussed the critical role of 3D culture systems in regulating cellular behavior, secretome composition, and EV production. Specifically, key biomimetic parameters—including stiffness, pore size, surface patterning, and biochemical signaling—within engineered hydrogels, electrospun scaffolds, 3D-printed constructs, and porous biomaterials were examined for their ability to recapitulate or enhance the regulatory functions of the native ECM. We further highlighted the potential of EVs as delivery vehicles and therapeutic agents for the treatment of various diseases.

Despite the rapid progress in this field, significant challenges remain before biomaterial-assisted EV production can be translated into routine clinical applications. Although numerous studies have demonstrated that engineered 3D microenvironments can alter EV yield, cargo composition, and biological function, the underlying molecular mechanisms remain incompletely understood. In particular, the complex interplay between biochemical and biomechanical signaling pathways makes it difficult to establish universal design principles capable of consistently producing homogeneous EV populations with predictable therapeutic properties. Future studies integrating biomaterial engineering with mechanobiology, systems biology, and high-throughput multi-omics analyses will be essential to elucidate these regulatory mechanisms.

Another major challenge lies in the standardization and quality control of therapeutic EV production. In the United States, the Food and Drug Administration (FDA) regulates stem cell-based therapeutics; however, comparable regulatory frameworks specifically governing EV-based therapeutics are still evolving [329]. While the Minimal Information for Studies of Extracellular Vesicles (MISEV) guidelines provide important recommendations regarding EV isolation, purification, characterization, and storage [330,331], additional international consensus is needed to establish standardized quality control criteria, potency assays, batch-to-batch reproducibility, and release specifications for clinical-grade EV products [332]. Such standardization will be critical for improving the safety, reproducibility, and regulatory approval of EV-based therapies.

Recent advances in EV bioengineering have also provided promising strategies to overcome several translational barriers. Hybrid vesicle systems, membrane functionalization through chemical conjugation, and genetic engineering of parental cells have significantly improved cargo loading efficiency, biodistribution, targeting specificity, and therapeutic efficacy while reducing manufacturing variability. Nevertheless, challenges related to large-scale production, purification efficiency, storage stability, immunogenicity, and manufacturing costs remain substantial. The continued optimization of bioreactor technologies, scalable purification methods, and good manufacturing practice (GMP)-compliant production pipelines will be indispensable for enabling widespread clinical translation [333].

Importantly, biomaterial-assisted 3D culture systems offer a promising solution to one of the greatest bottlenecks in EV therapeutics—low production yield. Compared with conventional 2D cultures, appropriately designed 3D microenvironments can better mimic native tissue architecture, support long-term cell viability and proliferation, and increase EV production while simultaneously modulating EV cargo and biological activity. As our understanding of the biomaterial-mediated regulation of EV biogenesis continues to improve, rationally engineered 3D culture platforms are expected to become increasingly powerful tools for producing standardized, high-quality therapeutic EVs. Ultimately, the convergence of biomaterials science, mechanobiology, EV engineering, and scalable manufacturing technologies will accelerate the translation of EV-based therapies from laboratory research to clinical practice.

## Figures and Tables

**Table 1:** Effects of scaffold stiffness on cell behavior and their underlying mechanisms.

Stiffness	Cell Type	Cell Behavior	Mechanism	Reference
High (17 kPa elastic modulus)	D1 murine MSCs	Osteogenic differentiation	High actomyosin contraction	[92]
Low (9 kPa elastic modulus)		Adipogenic differentiation	Low actomyosin contraction	
High (12 kPa ± 1.73 shear modulus)	Human MSCs	Higher expression of osteogenic genes	Polarized F-actin stress fibers	[102]
Low (1 kPa ± 0.16 shear modulus)		Lower expression of osteogenic genes	Diffuse F-actin stress fibers	
High (3 and 15 kPa elastic modulus)	Human MSCs	30% Faster cell proliferation	Increased p-ERK1/2 and YAP activity	[104]

**Table 2:** Effects of scaffold pore size on cell behavior and their underlying mechanisms.

Pore Size	Cell Type	Cell Behavior	Mechanism	Reference
Small (~100–300 µm^2^)	IC-21 murine macrophages	Elongated, spread morphology, high M1/M2 macrophage ratio	Small void space physically prevents aggregation and cell-to-cell interaction	[115]
	hBMSCs	Small cell aggregates (14.8 µm average aggregate diameter after 48 h)		
Large (~300–1700 µm^2^)	IC-21 murine macrophages	Round morphology, high M2/M1 macrophage ratio	Large void space physically allows aggregation and cell-to-cell interaction	
	hBMSCs	Large cell aggregates (32.0 µm average aggregate diameter after 48 h)		
Large (250–425 µm diameter range)	Murine BMSCs and SMSCs	Lower degree of stemness, more osteogenesis	High curvature angle increases cytoskeletal strain and YAP nuclear translocation	[114]
Small (60–125 µm diameter range)	Murine BMSCs and SMSCs	Higher degree of stemness, less osteogenesis	Lower curvature angle decreases cytoskeletal strain and YAP nuclear translocation	

**Table 3:** Effects of scaffold patterning on cell behavior and their underlying mechanisms.

Patterning	Cell Type	Cell Behavior	Mechanism	Reference
Linear grooves	C2C12 mouse myoblasts	Increased proliferation, myogenesis, and enhanced myotube alignment	Large widths of grooves and rough surface material trigger specific FAK and MAPK activation	[134]
Cubical pores (~830 µm pore diameters)	Whole unprocessed human bone marrow and hBMSCs	Osteogenic, adipogenic and chondrogenic protein expression	Cube geometry increased elastic modulus, larger pore size, higher porosity uniformity, and cell-to-cell interactions	[135]
Cylindrical pores (~730 µm pore diameters)		Adipogenic and chondrogenic protein expression	Cylindrical geometry decreased elastic modulus, pore size, porosity uniformity, and cell-to-cell interactions	
Rectangular microislands	WJ-MSCs	Neurogenesis	High aspect ratio to generate stretch-induced mechanotransduction with EPM to enhance Ca^2+^-related differentiation	[137]
Square microislands		Adipogenesis	Low aspect ratio with low stretch-induced mechanotransduction and/or EPM to trigger or enhance Ca^2+^-related differentiation	
Mesh-like	Ad-MSCs	Increased angiogenic and anti-inflammatory genes and cytokines, more M2 macrophages	Mesh topography directed cell shapes of both round and elongated morphologies with combinations of mechanotransduction and cell-to-cell interactions mechanotransduction and cell-to-cell interactions	[145]

**Table 4:** Effects of scaffold chemical signaling on cell behavior and their underlying mechanisms.

Chemical Signaling	Cell Type	Cell Behavior	Mechanism	Reference
High number of RGD adhesion sites	IC-21 murine macrophages	Elongated, spread morphology, high M1/M2 ratio	More integrin binding leads to more mechanotransduction	[115]
Low number of RGD adhesion sites		Round morphology, high M2/M1 macrophage ratio	Less integrin binding leads to less mechanotransduction	
High number of RGD adhesion sites	D1 murine MSCs	Osteogenic differentiation	More integrin stimulation caused more mechanotransduction	[92]
Low number of RGD adhesion sites		Adipogenic differentiation	Less integrin stimulation caused less mechanotransduction	
TGF-β	Human MSCs	Increased smooth muscle cell markers (α-actin and calponin-1) for cells seeded on stiff gels)	Activated Smad2/3 and stronger mechanotransduction signals working together	[104]
		Increased chondrogenic and adipogenic cell markers (collagen-II and LPL)	Activated Smad2/3 and weaker mechanotransduction signals working together	
Laminin	AT-MSCs	Neurogenic differentiation	Laminin stimulation of αvβ3 integrin	[142]
	BM-MSCs	Adipogenic differentiation	No laminin stimulation	

**Table 5:** Effects of hydrogels on cellular and EV responses.

Scaffold Composition	Design Parameters	Cell Type	Cellular and EV Responses	Biological Functions	Reference
Alginate	Elastic moduli decrease to ~3 kPa	Bone marrow aspirate hMSCs D1 murine MSCs (to observe functional changes)	EV output increased by 2-fold and 5-fold compared to stiff (~20 kPa) and plastic substrates, respectively	Lung edema and vascular permeability reduced more effectively than EVs from 2D plastic substrates	[191]
Polyacrylamide	High shear modulus (12 kPa ± 1.73)	hMSCs	Higher expression of osteogenic genes (Runx2, osterix, type I collagen, ALKP, and osteocalcin)	Not reported	[102]
	Low shear modulus (1 kPa ± 0.16)		Lower expression of osteogenic genes (Runx2, osterix, type I collagen, ALKP, and osteocalcin)		

**Table 6:** Effects of porous scaffolds on cellular and EV responses.

Scaffold Composition	Design Parameters	Cell Type	Cellular and EV Responses	Biological Functions	Reference
PLCL with HA nanoparticles	Smooth fibers	Rat BMSCs and RAW264.7 macrophages	Decreased expression of angiogenic, osteogenic, and immunomodulatory markers with less M2 macrophage polarization	Less effective bone regeneration	[202]
	Microporous fibers		Increased expression of angiogenic, osteogenic, and immunomodulatory markers with more M2 macrophage polarization	More effective bone regeneration	
PLLA	Small pore size (60–125 µm diameter range)	Murine BMSCs and SMSCs	Decreased expression of CTGF, YAP1, CD146, Runx2, and SP7	Not reported	[114]
			Higher expression of Gli1 and Col3 with lower expression of osteogenic markers		[204]
	Large pore size (250–425 µm diameter range)		Higher expression of CTGF, YAP1, CD146, Runx2, and SP7		[114]
			Lower expression of Gli1 and Col3 with higher expression of osteogenic markers		[204]

**Table 7:** Effects of electrospun and 3D printed scaffolds on cellular and EV responses.

Scaffold Composition	Design Parameters	Cell Type	Cellular and EV Responses	Biological Functions	Reference
PCL	Mesh patterning	Ad-MSCs	Higher expression of angiogenic paracrine factors (PGE2, iNOS, TGF-β, VEGF, and HGF) and anti-inflammatory M2 macrophage markers (IL-10 and Arg-1)	Neater scar in wound healing	[145]
PDMS	Aligned patterning	Human umbilical vein ECs	Higher expression of miR-143 and miR-145	Not reported	[224]
	Random patterning		Lower expression of miR-143 and miR-145		

**Table 8:** Effects of chemically modified scaffolds on cellular and EV responses.

Scaffold Composition	Design Parameters	Cell Type	Cellular and EV Responses	Biological Functions	Reference
Alginate	5-fold increase in RGD functionalization	Bone marrow aspirate hMSCs	2-fold decrease in EV number per cell	Lung edema and vascular permeability reduced	[191]
	5-fold reduction in RGD functionalization		2-fold increase in EV number per cell		
Non-coated 2D flask	CS-NO additive	hp-MSCs	Increased VEGF and miR-126 expression	Improved angiogenic activity in HUVECs and murine hind limb ischemia models	[230]
	No CS-NO		Decreased VEGF and miR-126 expression	Decreased angiogenic activity in HUVECs and murine hind limb ischemia models	

## Data Availability

No new data were created or analyzed in this study. Data sharing is not applicable to this article.
